# Accumulation of Ca_v_3.2 T-type Calcium Channels in the Uninjured Sural Nerve Contributes to Neuropathic Pain in Rats with Spared Nerve Injury

**DOI:** 10.3389/fnmol.2018.00024

**Published:** 2018-02-08

**Authors:** Wen Chen, Ye-Nan Chi, Xue-Jing Kang, Qing-Ying Liu, Hao-Lin Zhang, Zhi-Hua Li, Zi-Fang Zhao, Yin Yang, Li Su, Jie Cai, Fei-Fei Liao, Ming Yi, You Wan, Feng-Yu Liu

**Affiliations:** ^1^Neuroscience Research Institute, Peking University, Beijing, China; ^2^Department of Neurobiology, School of Basic Medical Sciences, Peking University, Beijing, China; ^3^Key Laboratory for Neuroscience, Ministry of Education/National Health and Family Planning Commission, Peking University, Beijing, China; ^4^Department of Anesthesiology, Dongfang Hospital, Beijing University of Chinese Medicine, Beijing, China; ^5^Department of Human Anatomy, School of Basic Medical Sciences, Zhengzhou University, Zhengzhou, China; ^6^Center of Medical and Health Analysis, Peking University, Beijing, China

**Keywords:** Ca_v_3.2 T-type calcium channels, uninjured nerve sensitization, mechanical allodynia, neuropathic pain, spared nerve injury

## Abstract

Injuries to peripheral nerve fibers induce neuropathic pain. But the involvement of adjacent uninjured fibers to pain is not fully understood. The present study aims to investigate the possible contribution of Ca_v_3.2 T-type calcium channels in uninjured afferent nerve fibers to neuropathic pain in rats with spared nerve injury (SNI). Aβ-, Aδ- and C-fibers of the uninjured sural nerve were sensitized revealed by *in vivo* single-unit recording, which were accompanied by accumulation of Ca_v_3.2 T-type calcium channel proteins shown by Western blotting. Application of mibefradil, a T-type calcium channel blocker, to sural nerve receptive fields increased mechanical thresholds of Aβ-, Aδ- and C-fibers, confirming the functional involvement of accumulated channels in the sural nerve in SNI rats. Finally, perineural application of mibefradil or TTA-P2 to the uninjured sural nerve alleviated mechanical allodynia in SNI rats. These results suggest that axonal accumulation of Ca_v_3.2 T-type calcium channels plays an important role in the uninjured sural nerve sensitization and contributes to neuropathic pain.

## Introduction

Peripheral neuropathic pain is characterized by spontaneous pain, mechanical allodynia and thermal hyperalgesia and results from lesions to the peripheral nervous system. Maladaptive plasticity, such as peripheral and central sensitization, is a core mechanism of neuropathic pain (Fields et al., 1998; Woolf and Salter, 2000; Latremoliere and Woolf, 2009; Anderson et al., 2017; Colloca et al., 2017). After attacked by external damage or multiple pathophysiological changes (Dubin and Patapoutian, 2010; Peleshok and Ribeiro-da-Silva, 2011), peripheral afferents produce spontaneous activities and ectopic discharges, and initiate central sensitization (including spinal sensitization).

On the other hand, adjacent uninjured afferents re-innervate the epidermis of injured fibers, and evoked activities in these uninjured afferents may induce evoked pain through sensitized spinal neurons (Ringkamp and Meyer, 2005; Shim et al., 2005; Campbell and Meyer, 2006; Duraku et al., 2013). In a mouse model of spared nerve injury (SNI), Aδ-mechanoreceptors and C-fibers in the spared sural nerve produce significantly more action potentials in response to suprathreshold mechanical stimulation (Smith et al., 2013). All these studies indicate possible contribution of the uninjured afferent sensitization to neuropathic pain.

Ion channels contribute to peripheral sensitization. For example, re-distribution of Na_v_1.8 in uninjured axons contributes to the generation of spontaneous activities of uninjured C-fibers and neuropathic pain (Gold et al., 2003). Recently, T-type calcium channels (also named low voltage activated calcium channels) have attracted much attention. T-type channels have been first well described functionally in primary sensory neurons (Carbone and Lux, 1984). Under physiological conditions, these channels are activated at near-resting membrane potentials, thereby regulating neuronal excitability (Perez-Reyes, 2003; Iftinca and Zamponi, 2009; Todorovic and Jevtovic-Todorovic, 2011). The pore-forming α1 subunit of T-type channels has at least three subtypes, α1G (Ca_v_3.1), α1H (Ca_v_3.2) and α1I (Ca_v_3.3). Ca_v_3.2 T-type channel proteins are dominantly expressed in small and medium-sized dorsal root ganglion (DRG) neurons, and unregulated in the DRG after nerve injury (Talley et al., 1999; Jagodic et al., 2008; Takahashi et al., 2010; Rose et al., 2013; Yue et al., 2013; Stemkowski et al., 2016; Li et al., 2017). In neuropathic rats, treatment with the antisense (AS) of Ca_v_3.2, but not Ca_v_3.1 or Ca_v_3.3, reduced neuropathic pain, suggesting that Ca_v_3.2 channels in DRG neurons possibly play roles in neuropathic pain (Bourinet et al., 2005, 2016). In addition to DRG neurons, Ca_v_3.2 T-type calcium channels are also expressed in peripheral sciatic nerves and hairy skin (Rose et al., 2013; François et al., 2015; Djouhri, 2016; Bernal Sierra et al., 2017). So, it is of interest to know whether Ca_v_3.2 T-type calcium channels in uninjured sural nerves participate in neuropathic pain.

The present study aims to investigate whether uninjured afferents fibers from the spared sural nerve are sensitized in SNI model of rats and their potential contribution to neuropathic pain. Our results showed that uninjured sural nerves were sensitized in SNI rats, and that accumulated Ca_v_3.2 T-type calcium channels in uninjured sural nerve contributed to peripheral sensitization and neuropathic pain.

## Materials and Methods

### Chemicals, Antibodies and Animals

Mibefradil (a T-type calcium channel antagonist; Sigma-Aldrich, St. Louis, MO, USA) was dissolved in normal saline (NS) to a 30 mM stock solution, stored at −20°C, and diluted to desired concentrations just before use. TTA-P2 (a novel, selective T-type calcium channel blocker; Alomone Labs, Jerusalem, Israel) was dissolved in dimethyl sulfoxide (DMSO) to a 10 mM stock solution, stored at −20°C, and diluted to desired concentrations just before use.

For immunofluorescence staining, polyclonal rabbit anti-Ca_v_3.2 antibody was obtained from Alomone Labs (Jerusalem, Israel). Alexa Fluor 488 goat anti-rabbit IgG (H+L) and Alexa Fluor 568 goat anti mouse IgG (H+L) were purchased from Invitrogen (Carlsbad, CA, USA).

For Western blot, polyclonal rabbit anti-Ca_v_3.2 antibody was obtained from Santa Cruz Biotechnology (Dallas, TX, USA). Monoclonal mouse anti-rat β-actin antibody and horseradish peroxidase (HRP)-conjugated secondary antibodies including goat anti-rabbit IgG and goat anti-mouse IgG were purchased from Santa Cruz Biotechnology (Dallas, TX, USA). All other chemicals or reagents were obtained from Sigma-Aldrich, Invitrogen, or Pierce except as mentioned in the text.

Adult male Sprague-Dawley rats weighing 200–250 g at the beginning of the experiment were provided by the Department of Experimental Animal Sciences, Peking University Health Science Center. Rats were housed 5 per cage in a temperature- and light-controlled room under a 12 h:12 h light:dark cycle with water and food available *ad libitum*. The animals were handled and habituated 3–5 days before all experiments. The experiments were carried out in accordance with the recommendations of the Guidelines of the International Association for the Study of Pain (Zimmermann, 1983). The protocol was approved by the Animal Care and Use Committee of Peking University (Permit Number: LA2012-76). The behavioral experimenters were kept blind from the grouping of the rats.

### SNI Model

The SNI model was established as previously described (Decosterd and Woolf, 2000) as in our lab (Zhang et al., 2016). After anesthetization with 1% sodium pentobarbital (0.5 ml/100 g, *i.p.*), the left tibial nerve and common peroneal nerve were tightly-ligated and sectioned distal to the ligation, leaving the sural nerve intact. Muscle and skin were closed in two layers. Sham-operation rats experienced exposure of the sciatic nerve and its branches but without any lesions.

### Behavioral Test for Mechanical Allodynia

Mechanical allodynia of the left hind paw was tested before and after surgery at different time points. The 50% paw withdrawal threshold (PWT) in response to a series of *von* Frey hairs (Stoelting, Wood Dale, IL, USA) was examined by the “up and down” method as described previously (Chaplan et al., 1994; Jiang et al., 2008; Liu et al., 2012; Deuis et al., 2017). Briefly, the rat was placed on a metal mesh floor covered with an inverted clear plastic cage (18 × 8 × 8 cm) and allowed a 15-min period for habituation. Eight *von* Frey hairs were chosen (0.4, 0.6, 1.0, 2.0, 4.0, 6.0, 8.0 and 15.0 g). Each trial started with a *von Frey* force of 2.0 g delivered perpendicularly to the plantar surface of the left hind-paw. An abrupt withdrawal of the foot during stimulation or immediately after the removal of the filament was recorded as a positive response. Once a positive or negative response was evoked, the next weaker or stronger filament was applied. This procedure was repeated until six stimuli after the first change in response had been observed. The 50% PWT was calculated using the following formula: 50% PWT = 10^ (X + kd)^/10^4^, where X is the value of the final *von Frey* filament used (in log units), k is a value measured from the pattern of positive/negative responses, and d is the average increment (in log units) between the *von* Frey hairs (Dixon, 1980). If a rat responded to the lowest *von* Frey hair, a value of 0.25 g was assigned; if a rat did not respond to the highest *von* Frey hair, the value was recorded as 15.0 g. Only those rats with 50% PWT less than 4.0 g were selected and used in subsequent experiments.

### *In Vivo* Single-Unit Recording

**Figure 1:**
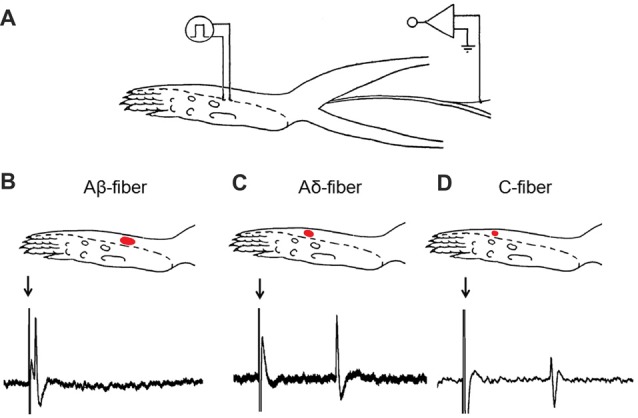
Experimental setup for *in vivo* single-unit recording in the sural nerve. **(A)** Recording site on the sural nerve and stimulating point on the receptive field. **(B–D)** Typical examples of the receptive field and the conduction velocity (CV) from Aβ-, Aδ- and C-fibers in Sham-operation rats. A red dot represents the receptive field determined by a *von* Frey filament with a force twice the response threshold of the fiber. As representative examples shown here, for the Aβ-fiber **(B)**, receptive field = 11 mm^2^, CV = 27.18 m/s (conduction distance = 56 mm; latency = 2.06 ms); for the Aδ-fiber **(C)**, receptive field = 8 mm^2^, CV = 2.62 m/s (conduction distance = 71 mm; latency = 27.07 ms); for the C-fiber **(D)**, receptive field = 5 mm^2^, CV = 1.76 m/s (conduction distance = 54 mm; latency = 30.70 ms). Arrows: stimulus artifacts.

*In vivo* single-unit recording was carried out 14 days after SNI or sham surgery. The rat was anesthetized with urethane (1.5 g/kg, *i.p.*) and the trachea was cannulated. Deep anesthesia was judged by absent eye-blink reflexes to air-puffs and withdrawal reflexes to noxious limb stimulation. An additional dose of urethane (100 mg/kg) was administered during the experiment when deemed necessary. The rat was placed on a water-perfused heating blanket to maintain core temperature (37 ± 0.5°C). As described previously, a midline incision was made from the mid-thigh to the ankle of the left hind limb to expose the sural nerve. The incised skin was retracted and formed a pool filled with warm paraffin oil over the exposed tissue. Under a dissecting microscope, the sural nerve was separated from the adjacent tissues and cut proximally at its junction with the sciatic nerve. For single-unit recording, the epineurium was first removed from the distal part of the cut end of the nerve, and bundles of nerve fibers were freed. Then, the perineurium was removed, and the fiber bundle was teased on a small mirror-based platform with sharpened forceps under a dissecting microscope until a fine nerve filament was isolated (Leem et al., 1993; Pogatzki et al., 2002). Recordings were made from the nerve filament that was placed on a platinum electrode with the reference electrode pinned to the nearby tissue (Figure 1A).

#### Identification of Aβ-, Aδ- and C-fiber in the Uninjured Sural Nerve

For each fiber, the identification procedure was as follows. First, the fiber was monitored for 2 min to assess spontaneous activities. Spontaneous discharges were defined as discharges greater than 1 spike/min (Ali et al., 1999; Wu et al., 2002).

Second, mechanical threshold was examined by applying punctate mechanical stimuli to the center of the receptive field with a set of calibrated *von* Frey hairs (0.008, 0.02, 0.04, 0.07, 0.16, 0.4, 0.6, 1.0, 1.4, 2.0, 4.0, 6.0, 8.0, 10.0, 15.0 and 26.0 g). The *von* Frey hair stimuli were applied with increasing force. Mechanical threshold was defined as the lowest force that elicited two or more spikes within 1 s, in at least 6 out of 10 trials. The *von* Frey hair was applied to the receptive field for 2 s per stimulus. The time interval between different intensities of the *von* Frey hair was no less than 10 s. Mechanical threshold was determined within 5 min (Chen and Levine, 2001). To avoid skin damage, the stiffest *von* Frey hair used in these studies was 26.0 g. If an afferent did not respond to 26.0-g *von* Frey hair, the next higher *von* Frey hair was regarded as threshold (60.0 g; Ringkamp et al., 2012).

Third, mechanical receptive field was mapped using a *von* Frey hair with a bending force approximately twice the individual response threshold. The receptive field was drawn on a diagram of the plantar hind paw, and the area was estimated by measuring the length and width of the receptive field on the drawing (Figures 1B–D). No detailed mapping of receptive field was made for fibers with response thresholds 26.0 g because this would have required repeated application of the 60.0-g filament, which could cause sensitization (Pogatzki et al., 2002).

Finally, to determine the conduction velocity (CV), the receptive field was stimulated with bipolar silver-wire stimulating electrodes. Constant current pulses (frequency: 0.5–1.0 Hz, duration: 0.5–2 ms) were delivered in gradually increasing intensities. CV was determined from the response latency and the distance between recording and stimulating electrodes. Fibers were classified as myelinated Aβ-fiber (CV ≥ 24 m/s), or thin myelinated Aδ-fiber (2 m/s ≤ CV < 24 m/s), or unmyelinated C-fiber (CV < 2 m/s) based on its CV (Djouhri and Lawson, 2004; Figures 1B–D). Single-unit discharge was captured and analyzed by a CED 1401 interface (Cambridge Electronic Design, Cambridge, UK) coupled to a Pentium computer with Spike 2 software.

#### Effects of Mibefradil on Mechanical Threshold of Single Fiber

To clarify whether T-type calcium channels were involved in sural nerve sensitization, mibefradil (0.01, 0.10 and 1.0 mM, 10 μl) was injected to the receptive field. The mechanical threshold of the single fiber was tested before, 30 and 60 min after drug application, respectively. To avoid any possible skin damages, the maximal threshold was set to 60.0 g (*von* Frey hair used was 26.0 g).

### Western Blot

The sural nerve was disrupted in the RIPA lysis buffer (Tris-HCl (pH 7.4) 50 mM, NaCl 150 mM, 1% NP-40, 0.1% SDS with protease inhibitor). The sample was sonicated on ice, and then centrifuged at 12,000 *g* for 15 min at 4°C to isolate the supernatant containing total proteins.

The protein sample was separated on a SDS-PAGE gel and transferred to a PVDF membrane. After blocking with 5% non-fat dried milk in TBST (20 mM Tris-HCl, 150 mM NaCl, 0.05% Tween 20, pH 7.6) buffer solution, the PVDF membrane was incubated with rabbit anti-Ca_v_3.2 (1:500), mouse anti-β-actin (1:2000) antibodies in TBST buffer with 5% non-fat dried milk overnight at 4°C. After washing with TBST buffer, the membrane was incubated with goat anti-rabbit antibody or goat anti-mouse antibody (HRP labeled) diluted with 5% non-fat dried milk in TBST and detected with ECL reagents (Amersham Biosciences, Arlington Heights, IL, USA). Blots were scanned with Spot Advanced and Photoshop 5.0 (Adobe, Inc.) softwares, and band densities were compared with TotalLAB software.

### Immunofluorescence Staining

As described in our previous study (Jiang et al., 2008), the rat was perfused with NS followed by 4% paraformaldehyde (PFA) in 0.1 M phosphate buffer (PB). The sural nerve was removed, post-fixed in 4% PFA for 4 h and dehydrated in 30% sugar solution. Seven days later, the sural nerve tissue was cut in 8-μm-thick serial longitudinal sections and mounted on a gelatin/chrome alum-coated glass slide. After blocking in normal goat serum, sural nerve sections were co-incubated with a combination of rabbit anti-Ca_v_3.2 polyclonal antibody (1:300, Alomone Labs) in 1.0% bovine serum albumin and 0.3% Triton-X100 in 0.01 mol/L phosphate-buffered saline (PBS) and one of the following antibodies: (1) mouse anti-rat neurofilament 200 (NF200, 1:1000, Sigma); (2) mouse anti-rat calcitonin gene-related peptide (CGRP, 1:1000, Sigma) over two nights at 4°C. After washing with PBS, sections were incubated with a mixture of Alexa Fluor 488 goat anti-rabbit IgG (H+L) and Alexa Fluor 568 goat anti-mouse IgG (H+L; 1:500; Invitrogen, Life Technologies™, USA) overnight at 4°C. The stained sections were captured with a fuorescence microscope (Leica, Germany).

### Effects of Mibefradil on Mechanical Threshold of SNI Rats

Mibefradil (0.10 and 1.0 mM, 100 μl) or TTA-P2 (0.05 and 0.5 mM, 100 μl) was injected perineurally to the sural nerve as follows. Under isoflurane anesthesia, the rat was held in lateral recumbency with the limb to be injected straighten. A 30-gauge injection needle was advanced from the ankle joint in parallel with the crus. Luminous Blue was used as a tracer to indicate the appropriate injection point. Sterile NS or 0.17% DMSO was used as a control.

### Data Analysis

Statistical analyses were performed with GraphPad Prism 5 for Windows (GraphPad Software, Inc., USA). All data were expressed as mean ± SEM. Two-tailed unpaired Student’s *t*-test was used for comparison of the mean values between two groups. One-way analysis of variance (ANOVA) followed by Tukey *post hoc* test or two-way ANOVA followed by Bonferroni *post hoc* test was used for multiple comparison. The Mann-Whitney U-test or the Kruskal-Wallis H-test was used for comparing independent groups with non-normal distributed variables. Differences with *p* < 0.05 were considered statistically significant.

## Results

### Mechanical Allodynia in SNI Rats

**Figure 2:**
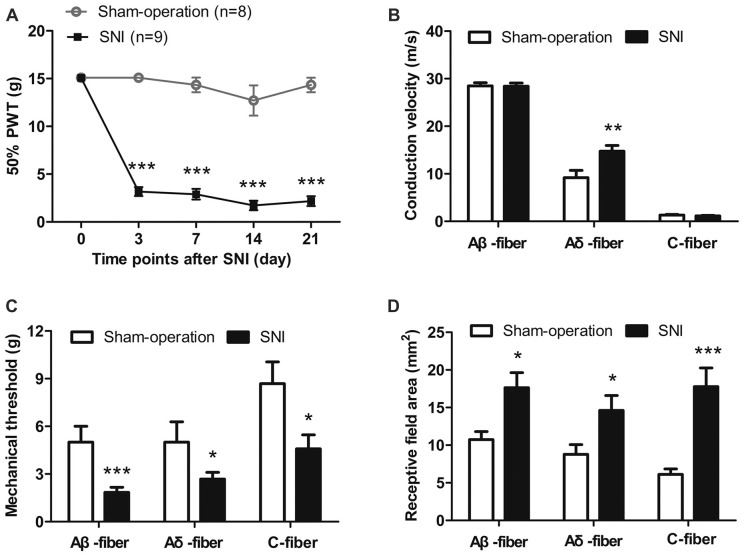
Mechanical allodynia and sensitization of uninjured sural nerve fibers in spared nerve injury (SNI) rats. **(A)** Mechanical allodynia in SNI rats 3–21 days after nerve injury. ***p < 0.001, two-way analysis of variance (ANOVA) with Bonferroni* post hoc* test, *n* = 8–9. **(B)** The CV of Aδ-fibers, but not Aβ-fibers and C-fibers was increased in SNI rats. **p < 0.01, unpaired *t* tests. **(C)** Mechanical thresholds of Aβ-, Aδ- and C-fibers were decreased in SNI rats. *p < 0.05, ***p < 0.001, unpaired *t* tests. **(D)** Receptive field areas of Aβ-, Aδ- and C-fibers were increased in SNI rats. *p < 0.05, ***p < 0.001, unpaired *t* tests.

As shown in Figure 2A, SNI rats exhibited mechanical allodynia 3–21 days after nerve injury. The 50% PWTs decreased significantly in SNI rats compared with those in sham-operation rats at day 3 (3.17 g ± 0.47 g vs. 15.10 g ± 0.00 g), day 7 (2.89 g ± 0.56 g vs. 14.34 g ± 0.76 g), day 14 (1.72 ± 0.50 g vs. 12.71 ± 1.58 g) and day 21 (2.17 g ± 0.52 vs. 14.34 ± 0.76 g; all *p* < 0.001, two-way ANOVA, *n* = 8–9). Mechanical thresholds of the contralateral hind paws remained unchanged (data not shown).

### Sensitization of the Uninjured Sural Nerve in SNI Rats

We investigated the characteristics of Aβ-, Aδ- and C-fibers in the uninjured sural nerves on day 14 after SNI or sham operation. A total of 25 Aβ-fibers, 22 Aδ-fibers and 24 C-fibers were identified from 33 sham-operation rats, and 53 Aβ-fibers, 45 Aδ-fibers and 21 C-fibers from 59 SNI rats.

#### Spontaneous Discharges of Sural Nerves

We first observed the percentage of sural nerve fibers that showing spontaneous discharges. 1/25 Aβ-fibers (4.0%), 1/22 Aδ-fibers (4.55%) and 1/24 C-fibers (4.17%) showed spontaneous discharges in the Sham-operation group. Similar results were observed in the SNI group (Aβ-fibers: 8/53, 15.09%; Aδ-fibers: 5/45, 11.11%; C-fibers: 2/21, 9.52%; *p* > 0.05, *χ*^2^ test).

In those sural nerve fibers showing spontaneous discharges, we further examined their frequencies. Frequencies of spontaneous Aβ-, Aδ- and C-fibers in Sham-operation group were 0.24 Hz, 0.97 Hz and 1.20 Hz, respectively, and in SNI group were 13.61 ± 3.34 Hz (range, 0.6–29.83 Hz), 3.81 ± 3.25 Hz (range, 0.17–16.8 Hz) and 5.95 ± 5.23 Hz (range, 0.72 and 11.17 Hz), respectively.

#### Increased CV of Aδ-fibers in SNI Rats

As shown in Figure 2B, the CVs of Aδ-fibers were significantly faster in SNI group than those in Sham-operation group (14.74 ± 1.21 m/s vs. 9.18 ± 1.54 m/s, *p* < 0.01, unpaired *t* tests). By contrast, the CVs of Aβ- and C-fibers in the SNI group remained unchanged (Aβ-fibers, 28.41 ± 0.66 m/s vs. 28.47 ± 0.69 m/s; C-fibers, 1.14 ± 0.13 m/s vs. 1.33 ± 0.11 m/s; *p* > 0.05, unpaired *t* tests).

#### Decreased Mechanical Thresholds and Increased Receptive Field Areas of Sural Nerve Fibers in SNI Rats

The mechanical response thresholds of Aβ-fibers were significantly lower in SNI group than those in Sham-operation group (1.85 ± 0.32 g vs. 5.01 ± 1.00 g;* p* < 0.001, unpaired *t* tests). A similar decrease was observed in both Aδ-fibers (2.68 ± 0.42 g vs. 5.01 ± 1.28 g, *p* < 0.05, unpaired *t* tests) and C-fibers (4.58 ± 0.88 g vs. 8.68 ± 1.37 g, *p* < 0.05, unpaired *t tests*; Figure 2C).

Mechanical receptive field areas were observed in SNI rats and in Sham-operation rats. For Aβ-fibers, the receptive field areas were significantly larger in SNI rats than those in Sham-operation rats (17.63 ± 2.02 vs. 10.73 ± 1.07 mm^2^, *p* < 0.05, unpaired*t tests*). Similarly, an increase was also observed in both Aδ-fibers (14.62 ± 1.97 vs. 8.77 ± 1.31 mm^2^, *p* < 0.05, unpaired *t* tests) and C-fibers (17.79 ± 2.46 mm^2^ vs. 6.12 ± 0.71 mm^2^, *p* < 0.001, unpaired *t* tests; Figure 2D).

These data suggest that Aβ-, Aδ- and C-fibers of the uninjured sural nerve in SNI rats are all sensitized and develop hypersensitivity to mechanical stimulation.

### Accumulation of Ca_v_3.2 T-type Calcium Channels in the Sural Nerve after SNI

**Figure 3:**
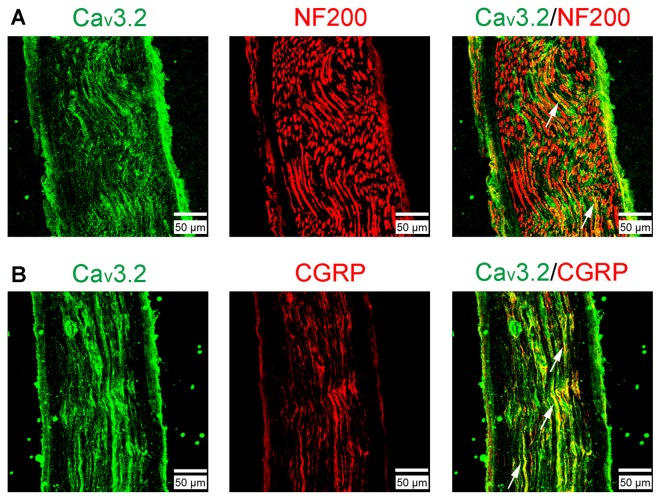
The expression of Ca_v_3.2 T-type calcium channels examined by immunofluorescent double staining in the sural nerve. **(A)** Ca_v_3.2 and neurofilament 200 (NF200). **(B)** Ca_v_3.2 and calcitonin gene-related peptide (CGRP). Scale bar, 50 μm. Arrows indicate some examples of the double-labeled axons.

Ca_v_3.2 T-type calcium channel proteins in sural nerves were observed in normal rats with immunofluorescence staining (Figure 3). Ca_v_3.2 channel protein immunoreactivity was present in partial NF200^+^ axons (a marker for myelinated fibers; Figure 3A), whereas Ca_v_3.2 channel immunoreactivity axons were highly co-expressed with CGRP (a marker for nociceptive peptidergic fibers; Figure 3B). Together, these results demonstrated the expression of Ca_v_3.2 channels were mainly in nociceptive peptidergic C-fibers and partially in myelinated fibers.

**Figure 4:**
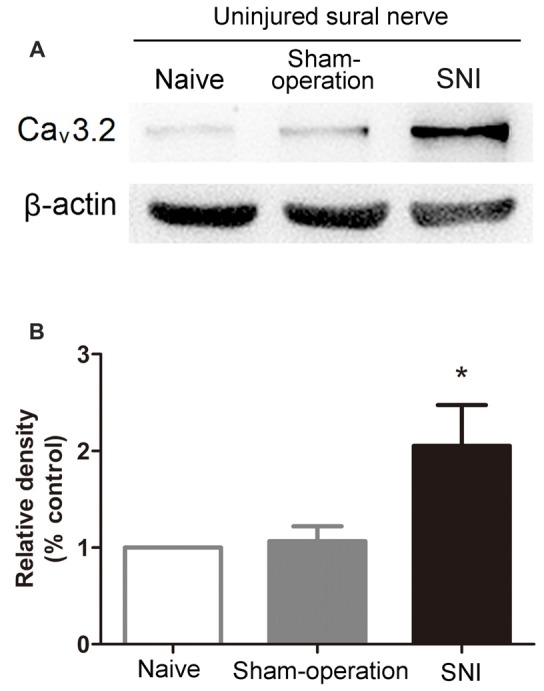
Increased expression of Ca_v_3.2 T-type calcium channel proteins in the uninjured sural nerve after SNI. **(A)** Representative Western blot bands of Ca_v_3.2 T-type calcium channel proteins (molecular weight: 260 kDa) 14 days after SNI. **(B)** Statistical analysis of the relative band densities of Ca_v_3.2 protein. β-actin is used as an internal control. Note that the expression of Ca_v_3.2 T-type calcium channel protein in ipsilateral sural nerve increased in SNI rats compared with Sham-operation rats. *p < 0.05, compared with the Sham-operation group, one-way ANOVA followed by Tukey* post hoc* test, *n* = 7.

We next examined the alteration of Ca_v_3.2 T-type calcium channel protein expression in sural nerves in SNI rats using Western blot assay. As expected, the expression of Ca_v_3.2 channel proteins in ipsilateral sural nerve was significantly increased 14 days in SNI rats compared with that in Sham-operation rats. Representative results of Western blot were shown in Figures 4A,B (2.05 ± 0.42 vs. 1.07 ± 0.16, *p* < 0.05, one-way ANOVA, *n* = 7).

These results indicate an accumulation of Ca_v_3.2 T-type calcium channels in sural nerves after SNI.

### Ca_v_3.2 T-type Calcium Channels Contribute to the Sensitization of the Uninjured Sural Nerve after SNI

To explore potential contribution of the accumulated Ca_v_3.2 T-type calcium channels to the sensitization of the sural nerve, we investigated the effect of mibefradil, a T-type calcium channel blocker, on mechanical threshold of single fiber in SNI rats. Mibefradil (0.01, 0.10 and 1.0 mM, 10 μl) was injected to the receptive field of the recorded fiber. The mechanical thresholds of single fibers were examined before, and 30 and 60 min after mibefradil application. The baseline thresholds before mibefradil application were different between the Sham-operation and SNI groups. The statistical results have been shown in Figure 2C.

**Figure 5:**
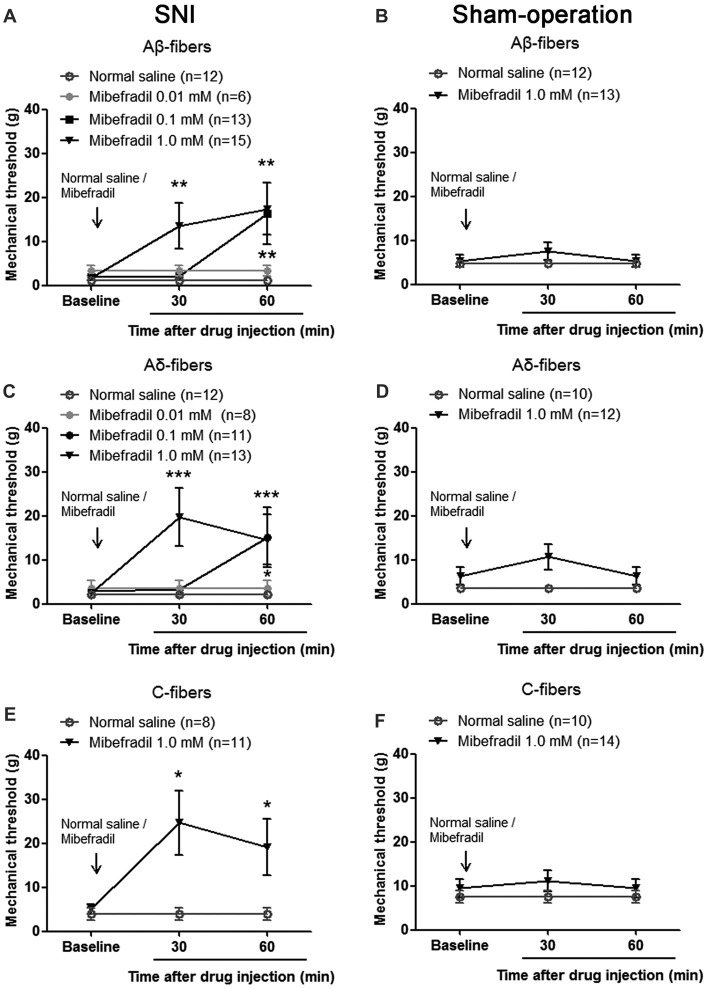
Mibefradil, a T-type calcium channel blocker, decreased mechanical thresholds of Aβ-, Aδ- and C-fibers in SNI rats. **(A)** Mibefradil (0.01, 0.10 and 1.0 mM, 10 μl) dose-dependently increased mechanical thresholds of Aβ-fibers in SNI rats. **p < 0.01, Kruskal-Wallis H-test, *n* = 6 –15. **(B)** Mibefradil (1.0 mM, 10 μl) had no effect on the mechanical thresholds of Aβ-fibers in Sham-operation rats. *n* = 12–13. **(C)** Mibefradil (0.01, 0.10 and 1.0 mM, 10 μl) dose-dependently increased mechanical thresholds of Aδ-fibers in SNI rats. *p < 0.05, ***p = 0.001, Kruskal-Wallis H-test, *n* = 8–13. **(D)** Mibefradil (1.0 mM, 10 μl) had no effect on mechanical thresholds of Aδ-fibers in Sham-operation rats. *n* = 10–12. **(E)** Mibefradil (1.0 mM, 10 μl) increased mechanical thresholds of C-fibers in SNI rats. *p < 0.05, Mann-Whitney U-test, *n* = 8–11. **(F)** Mibefradil (1.0 mM, 10 μl) had no obvious effect on mechanical thresholds of C-fibers in Sham-operation rats. *n* = 10–14.

As shown in Figure 5A, in SNI rats, mibefradil dose-dependently increased mechanical thresholds of Aβ-fibers. Mechanical thresholds increased significantly in 1.0-mM mibefradil-treated group (13.51 ± 5.14 g and 17.43 ± 5.91 g at 30 and 60 min after mibefradil application, respectively), and in 0.10-mM mibefradil-treated group (16.35 ± 6.94 g at 60 min) compared with those in the NS group (1.18 ± 0.40 g and 1.18 ± 0.40 g at 30 and 60 min, respectively; *p* < 0.01, Kruskal-Wallis H-test, *n* = 6–15). However, even at the highest concentration (1.0 mM), mibefradil had no obvious effects on mechanical thresholds of Aβ-fibers in Sham-operation rats (Figure 5B).

A similar change was observed in Aδ-fibers and C-fibers. Mechanical thresholds of Aδ-fibers increased significantly in 1.0-mM mibefradil-treated group (19.65 ± 6.60 g and 14.62 ± 5.67 g at 30 and 60 min after mibefradil application, respectively), and in 0.10-mM mibefradil-treated group (15.18 ± 6.82 g at 60 min) compared with those in NS group (2.14 ± 0.55 g and 2.14 ± 0.55 g at 30 and 60 min, respectively; *p* < 0.05 or *p* = 0.001, Kruskal-Wallis H-test, *n* = 8–13, Figure 5C). However, even at the highest concentration (1.0 mM), mibefradil had no obvious effects on mechanical thresholds of Aδ-fibers in Sham-operation rats (Figure 5D).

Because the sural nerve was relatively fine, it was difficult to record single C-fiber activity *in vivo*. So, we only examined the effect of 1.0 mM mibefradil on mechanical thresholds of C-fibers, and did not observe the effects of low dose of mibefradil (0.01 and 0.10 mM). In SNI rats, mechanical thresholds of C-fibers increased significantly in 1.0-mM mibefradil-treated group (24.64 ± 7.24 g and 19.09 ± 6.39 g at 30 and 60 min, respectively) compared with those in NS group (3.93 ± 1.38 g and 3.93 ± 1.38 g at 30 and 60 min, respectively; *p* < 0.05, Mann-Whitney U-test, *n* = 8–11, Figure 5E). Again, the highest concentration (1.0 mM) of mibefradil had no obvious effects in Sham-operation rats (Figure 5F).

These results suggest that the accumulated T-type calcium channel proteins are involved in uninjured sural nerve sensitization after SNI and that mibefradil decreases peripheral sensitization.

### Involvement of Ca_v_3.2 T-type Calcium Channels in Mechanical Allodynia after SNI

**Figure 6:**
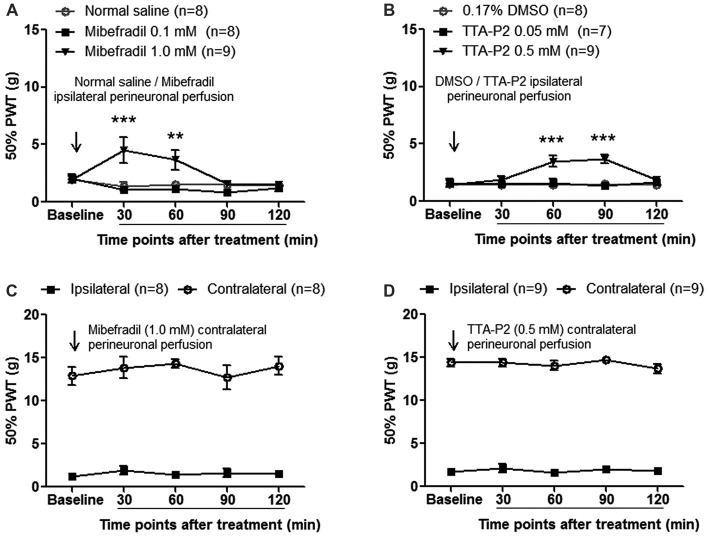
Perineural application of mibefradil or TTA-P2 decreased mechanical allodynia at day 14 after SNI. **(A,B)** Ipsilateral perineural perfusion of mibefradil (1.0 mM, 100 μl) or TTA-P2 (0.5 mM, 100 μl) partially reversed mechanical allodynia in a time-dependent manner. **p < 0.01, ***p < 0.001, two-way ANOVA followed by Bonferroni* post hoc* test, *n* = 7–9. **(C,D)** Contralateral perineural perfusion of mibefradil (1.0 mM, 100 μl) or TTA-P2 (0.5 mM, 100 μl) had no obvious effect on ipsilateral and contralateral mechanical thresholds. *n* = 8–9.

To investigate whether accumulated T-type calcium channels in sural nerves contributed to neuropathic pain, we first evaluated the effects of perineural application of mibefradil (0.10 and 1.0 mM, 100 μl) on mechanical allodynia in SNI rats. As shown in Figure 6A, at day 14 after SNI, mechanical thresholds increased significantly in 1.0-mM mibefradil-treated group (4.53 ± 1.14 g and 3.67 ± 0.85 g at 30 and 60 min after mibefradil application, respectively) compared with those in 0.10-mM mibefradil-treated group (1.04 ± 0.28 g and 1.15 ± 0.27 g at 30 and 60 min, respectively) and in NS group (1.35 ± 0.36 g and 1.55 ± 0.28 g at 30 and 60 min, respectively; *p* < 0.01 or 0.001, two-way ANOVA, *n* = 8–9).

Next, we evaluated the effects of perineural application of TTA-P2 (another novel, selective T-type calcium channel blocker) on mechanical allodynia in SNI rats. As shown in Figure 6B, at day 14 after SNI, mechanical thresholds were significantly increased in 0.5-mM TTA-P2-treated group (3.50 ± 0.46 g and 3.69 ± 0.40 g at 60 and 90 min after TTA-P2 application, respectively) compared with those in 0.05-mM TTA-P2-treated group (1.62 ± 0.34 g and 1.35 ± 0.28 g at 60 and 90 min, respectively) and in DMSO group (1.40 ± 0.21 g and 1.48 ± 0.25 g at 60 and 90 min, respectively; *p* < 0.001, two-way ANOVA, *n* = 7–9).

To exclude the possibility of the systemic effects of mibefradil or TTA-P2, a control experiment was carried out in which mibefradil or TTA-P2 at the same concentration was applied into the contralateral (right) side of SNI rats. No obvious effects were observed in ipsilateral or contralateral hind paws (Figures 6C,D), arguing against any systemic effects by mibefradil or TTA-P2.

These results suggest that accumulated Ca_v_3.2 T-type calcium channels in the uninjured sural nerve contribute to mechanical allodynia after SNI.

## Discussion

In the present study, by using *in vivo* single-unit recording technique, we show for the first time to our knowledge that the uninjured sural nerve is sensitized in SNI rats. More interestingly, Ca_v_3.2 channel proteins are accumulated in the uninjured sural nerve after SNI and contribute to peripheral sensitization and mechanical allodynia in neuropathic pain.

### Sensitization of Uninjured Sural Nerve Fibers in SNI Rats

Our single-unit recording experiments demonstrate peripheral sensitization of Aβ-, Aδ- and C-fibers in the uninjured sural nerve in SNI (Figure 2). These are supported not only by mechanical threshold reduction and receptive field expansion in Aβ-, Aδ- and C-fibers, but also by CV increases in Aδ-fibers.

Previous studies reported that peripheral nociceptors innervated by the uninjured nerve fibers were sensitized in spinal nerve ligation (SNL) rats (Shim et al., 2005). In sciatic nerve crush rats, uninjured saphenous nerve terminals extended onto the plantar surface of the foot, well beyond their limits in intact rats (Devor et al., 1979).

It has been proposed that Wallerian degeneration of injured nerves may explain the sensitization of adjacent uninjured afferent fibers in SNL models (Wu et al., 2002). However, unlike L5 SNL, Wallerian degeneration should not be a major factor in the SNI model because the SNI involves minimal co-mingling of uninjured and injured fibers (Decosterd and Woolf, 2000). Therefore, different mechanisms likely contribute to the uninjured afferent sensitization in SNI models. Possible mechanisms include nerve growth factor (NGF) and tumor necrosis factor-α (TNF-α) released from immune and epidermal cells in the target tissue of the injured afferents (Mearow et al., 1993; Harper et al., 1999; Koltzenburg et al., 1999; Meyer and Ringkamp, 2008; Kelleher et al., 2017). Adjacent uninjured afferent fibers gradually sprout into denervated skin territories (Devor, 1999; Duraku et al., 2012, 2013), where NGF and TNF-α lead to the sensitization of uninjured fibers (Harper et al., 1999; Leung and Cahill, 2010). Another possible mechanism is paracrine effects. There is co-mingling of injured and uninjured cell bodies in the L4 and L5 DRG in SNI rats. Injured cells produce paracrine molecules that act on uninjured cells to change their excitability. In addition, sciatic nerve injury induces macrophage infiltration into the DRG, which produces inflammatory mediators and neurotrophins and leads to non-injured neuronal sensitization (Kwon et al., 2013). Our preliminary data show increased excitability of uninjured DRG neurons (data not shown). We speculate that the excited uninjured neurons may transport sodium and calcium channels to the corresponding peripheral nerves, and induce the uninjured fiber sensitization (Gold et al., 2003; Jiang et al., 2008).

In the present study, there was no difference in the percentage of Aβ-, Aδ- and C-fibers that exhibited spontaneous activities between SNI and Sham-operation groups. Smith et al. (2013) also observed similar proportion of uninjured A- or C-fibers that showed spontaneous activities in SNI mice. We should note that in L5 SNL rats, uninjured L4 fibers may develop spontaneous firings, which result from Wallerian degeneration of the injured nerve fibers (Ali et al., 1999; Wu et al., 2002). By contrast, one explanation for the lack of increased percentage of spontaneous firings in uninjured fibers in SNI may be the absence of co-mingling of injured and uninjured fibers.

### Role of Axonal Ca_v_3.2 Channel Accumulation in the Sural Nerve Sensitization

The mechanism involved in the generation of uninjured afferent sensitization is not fully understood. It is well known that ion channels contribute to uninjured afferent sensitization. For example, the re-distribution of Na_v_1.8 plays an important role in generating of spontaneous activities of uninjured C-fibers (Gold et al., 2003). In the present study, we showed for the first time an increase in the expression of Ca_v_3.2 channels in the uninjured sural nerve after SNI (Figure 4).

Recently, with Ca_v_3.2-EGFP knock-in (KI) mouse, Ca_v_3.2 channel proteins were found to express within sciatic nerves (Rose et al., 2013; François et al., 2015; Djouhri, 2016; Bernal Sierra et al., 2017). Ca_v_3.2 axonal localization has two patterns: confined in the node of Ranvier of low-threshold mechanoreceptor (LTMR) in Aδ fibers (Aδ-LTMRs), and with a diffuse distribution along thin unmyelinated C-LTMRs. Ca_v_3.2 affects Aδ- and C-LTMR excitability under physiological conditions (Wang and Lewin, 2011; François et al., 2015). In our present study, we confirmed that Ca_v_3.2 channels were highly co-expressed with CGRP. Interestingly, Ca_v_3.2 T-type calcium channels were present in partial NF200^+^ axons (Figure 3). In classical view, DRG neurons are usually divided neurochemically into NF200^+^, CGRP^+^, et al. In fact, by recent reports, DRG neuron classification is much more complex (Usoskin et al., 2015; Li et al., 2016). There are several subpopulations of NF200^+^ neurons. So, in our experiment, Ca_v_3.2 was found to co-localize with a part, not all, NF200^+^ neurons, suggesting that Ca_v_3.2 is expressed in a subpopulation of NF200^+^ (large) neurons. Together, these results indicate that there are at least two populations of Ca_v_3.2 expressing sural nerve (peptidergic C-fiber and Aδ-LTMR) in rats, and provide a neuronal basis for Ca_v_3.2 channel modulation of peripheral sensitization and pain. Using a viral reporter approach, it was found that Ca_v_3.2 T-type channels were co-expressed with NF200 and CGRP in adult DRG neurons. Combining with our study, these results indicate axonal transport of Ca_v_3.2 T-channels from DRG cell bodies to peripheral axon.

Furthermore, we found increased Ca_v_3.2 protein expression in uninjured sural nerve after SNI (Figure 4). Considering that T-type calcium channels are activated before sodium channels, enhanced Ca_v_3.2 channels are well positioned for lowering the excitability threshold and facilitating action potential generation. This is verified in our study: T-type channel inhibition in receptive fields increased mechanical thresholds of Aβ-, Aδ- and C-fibers in the uninjured sural nerve (Figure 5). Therefore, our results suggest that increased Ca_v_3.2 channel proteins play important roles in the sensitization of the uninjured sural nerve after SNI.

Previous studies have shown that Ca_v_3.2 channels are rarely expressed in large Aβ-neurons and Aβ-fibers in mice and rats (Talley et al., 1999; Wang and Lewin, 2011; Rose et al., 2013; Bourinet et al., 2016). An interesting finding in our study is that Ca_v_3.2 channels participated in Aβ-fiber sensitization in the uninjured sural nerve after SNI (Figure 5A). These results suggest that a possible re-distribution of Ca_v_3.2 channel proteins after SNI (Bourinet et al., 2016). We speculate that a shift of Ca_v_3.2 channels from small and medium-sized DRG neurons to large ones occurs after SNI. Furthermore, the uninjured large Aβ-neurons may transport Ca_v_3.2 channels to peripheral nerve fibers, and thus induce Aβ fiber sensitization.

The sensitization of uninjured sural nerve was also characterized by increased CVs in Aδ-fibers (Figure 2B). The speed of action potential propagation in myelinated axons is affected mainly by axonal diameter, myelin thickness, and sodium channel clustering at nodes of Ranvier (Rasband and Peles, 2015; Fontaine et al., 2017; Nelson and Jenkins, 2017). Besides sodium channels, Ca_v_3.2 channels are also confined in the node of Ranvier of Aδ-LTMRs (François et al., 2015). Pharmacological experiments have confirmed T-type calcium channels as a predominant mechanism of calcium influx at nodes of Ranvier (Zhang and David, 2016; Fontaine et al., 2017). Mibefradil increased the latency of Aδ-LTMRs to repetitive mechanical stimulation (Shin et al., 2003). Based on these findings, Ca_v_3.2 channels are expected to regulate action potential conduction. We observed that the CV of the Aδ-fiber was significantly faster in the SNI group. Considering the increased Ca_v_3.2 protein expression in uninjured sural nerves, we suggest that Ca_v_3.2 channel may play a role in CV increase of uninjured Aδ-fibers after SNI.

Spontaneous activities of peripheral nerve play an important role in spontaneous pain (Wu et al., 2002). In our preliminary experiment, we found that mibefradil decreased spontaneous activities in Aβ- and Aδ-fibers in SNI rats. Although the number of fibers was only one respectively that we did not do a statistic, the results suggested that mibefradil may normalize the spontaneous activities.

### Role of Accumulated Axonal Ca_v_3.2 Channels in Neuropathic Pain

Sensitization of uninjured afferent nerve fibers may play an important role in neuropathic pain (Campbell and Meyer, 2006). We hypothesized that accumulated Ca_v_3.2 channels in uninjured sural nerve fibers contributed to neuropathic pain. Perineural application with mibefradil alleviated mechanical allodynia (Figure 6A). The contralateral administration of mibefradil did not influence the mechanical allodynia in the ipsilateral injured hind paw or in the contralateral uninjured hind paw (Figure 6C), which help exclude the possibility of systemic and central effects. Consistent with our hypothesis, local injection of mibefradil into planta pedis of injured hind paw, affected only the intact fibers, reversed neuropathic pain in SNL rats (Dogrul et al., 2003). Since mibefradil is a nonspecific T-type calcium channel blocker (Bourinet et al., 2016), we further observed the effects of TTA-P2, another novel and selective T-type calcium channel blocker. Consistent with mibefradil, TTA-P2 also reduced mechanical allodynia (Figure 6B). Considering the specificity of mibefradil and TTA-P2 on Ca_v_3.2 channels, further studies using subtype specific blockers or knockout mice will provide more evidence to this issue. Other subtypes of calcium channels, like Ca_v_3.1 and Ca_v_3.3 which are not excluded in the present study, need further investigation.

Taken together, accumulated Ca_v_3.2 channels in the uninjured sural nerve contribute to mechanical allodynia after SNI.

### Possible Mechanisms of Ca_v_3.2 Channel Accumulation in the Uninjured Sural Nerve

One possible molecular mechanism underlying Ca_v_3.2 channel accumulation in the uninjured sural nerve is inflammatory mediators (such as interleukin-6 or interleukin-1β) released from activated macrophages and satellite glial cells. Their combination to corresponding receptors upregulates the Ca_v_3.2 channels in uninjured DRG neurons (Kwon et al., 2013; Buckley et al., 2014; Weaver et al., 2015; Stemkowski et al., 2017). Another possibility may be similar to the situation in the IGF-1 (Li et al., 2014) or NGF-regulation on peripheral TRPV1 channels (Ji et al., 2002). NGF, released from immune and epidermal cells in the injured skin, may be absorbed by uninjured nerve fibers and then modulates the Ca_v_3.2 channel expression and translocation to peripheral axons.

## Conclusion

Our study suggests that accumulated Ca_v_3.2 channels in the uninjured sural nerve play an important role in peripheral sensitization and contribute to neuropathic pain. Ca_v_3.2 channels may be a potential peripheral target for the treatment of neuropathic pain.

## Author Contributions

WC, FL and YW designed the experiments; WC, YC, XK, QL, HZ, ZL, ZZ, YY and LS performed the experiments; JC, FL and MY analyzed the data; WC, YC, FL and YW wrote the manuscript.

## Conflict of Interest Statement

The authors declare that the research was conducted in the absence of any commercial or financial relationships that could be construed as a potential conflict of interest.

## Abbreviations

ANOVA: analysis of variance
AS: antisense
CGRP: calcitonin gene-related peptide
CV: conduction velocity
DMSO: dimethyl sulfoxide
DRG: dorsal root ganglion
HRP: horseradish peroxidase
KI: knock-in
LTMR: low-threshold mechanoreceptor
NF200: neurofilament 200
NGF: nerve growth factor
NS: normal saline
PB: phosphate buffer
PBS: phosphate-buffered saline
PFA: paraformaldehyde
PWT: paw withdrawal threshold
SNI: spared nerve injury
SNL: spinal nerve ligation
TNF-α: tumor necrosis factor-α.
